# Supplementary material for: Differential patch-leaving behavior during probabilistic foraging in humans and gerbils
Source: Commun Biol. 2024 Aug 15;7:1000. doi: 10.1038/s42003-024-06683-8 (PMC11327252; doi:10.1038/s42003-024-06683-8)
Supplement: Supplementary file 2 — Supplementary Information [file 42003_2024_6683_MOESM2_ESM.pdf]

Supplementary material

S1. Animals showed a decreasing task commitment within single sessions

The task behavior our animals showed in the probabilistic foraging task is likely susceptible to slow and steady changes in behavior throughout a single experimental session. Although it was ensured that the animals would start a session hungry and motivated, this motivation may decrease as a function of satiation, resulting in a decreased task commitment which conversely would increase the frequency of task-unrelated behavior. The latter would particularly impact behavioral parameters such as the average collection rate. To test this hypothesis, we conducted a within-session split-half analysis and compared the gerbils' behavioral parameters between the two session parts and for each level of patch-quality. For this purpose, we split each session into a first and a second half and tested for behavioral changes between the two splits for each patch quality using a 2x3 ANOVA with session split (first half versus second half of an experimental session) and patch quality as repeated measure factors.

The inspection of residence times showed a significant increase in the second compared to the first part of the session, as indicated by the significant main effect of session split [$F(1,16) = 23.776$, $p < .001$]. Also the average travel times associated with moving between the two spouts was significantly increased in the second half of a session (see Supplementary Figure 1 a & b), [$t(17) = -3.60$, $p = .002$]. Yet, whereas travel and residence times increased, there was no such effect on attempted reward captures (i.e., nose pokes) [$F(1,15) = 0.080$, $p = .781$] (see Supplementary Figure 1 c), but the intervals between single pokes increased significantly [$F(1,16) = 73.232$, $p < .001$] (see Supplementary Figure 1 d). Altogether this pattern of results suggests a decrease of the gerbils' behavioral efficiency with increasing session duration. As animals continued foraging, each further reward capture likely contributed to their steady satiation decreasing the animals' motivation. Consequently, being less motivated to perform the task, the animal became more and more distracted and the frequency of task-irrelevant actions (e.g. grooming, sniffing etc.) increased in the second compared to the first part of a session, leading to prolonged residences and travel times as well as inter-poke intervals while the frequency of goal-directed behavior itself, i.e., the number of nose pokes remained constant. Spending more time in patches of higher quality is worthwhile and resulted in higher yields per patch in the second half of a session in medium- and high-quality patches, but it was detrimental to the average yields in low-quality patches in which it is best to spend as little time as possible (see Supplementary Figure 1 e)). This interaction between patch quality and session split on the number of obtained rewards [$F(2,34) = 14.271$, $p < .001$] showed that the increases in residence times were not realized in order to optimize the foraging, but were rather a mere product of an increasing 'task-laziness' i.e., a diminished commitment to the task in the second half of the experiment leading to more reward gains in patches of higher quality as well as to average reward losses in patches of poor quality. The diminishing task commitment consequently led to a significant drop in

the average collection rate in the second compared to the first session split [$t(17) = 1.750$, $p = .045$] (see Supplementary Figure 1 f).

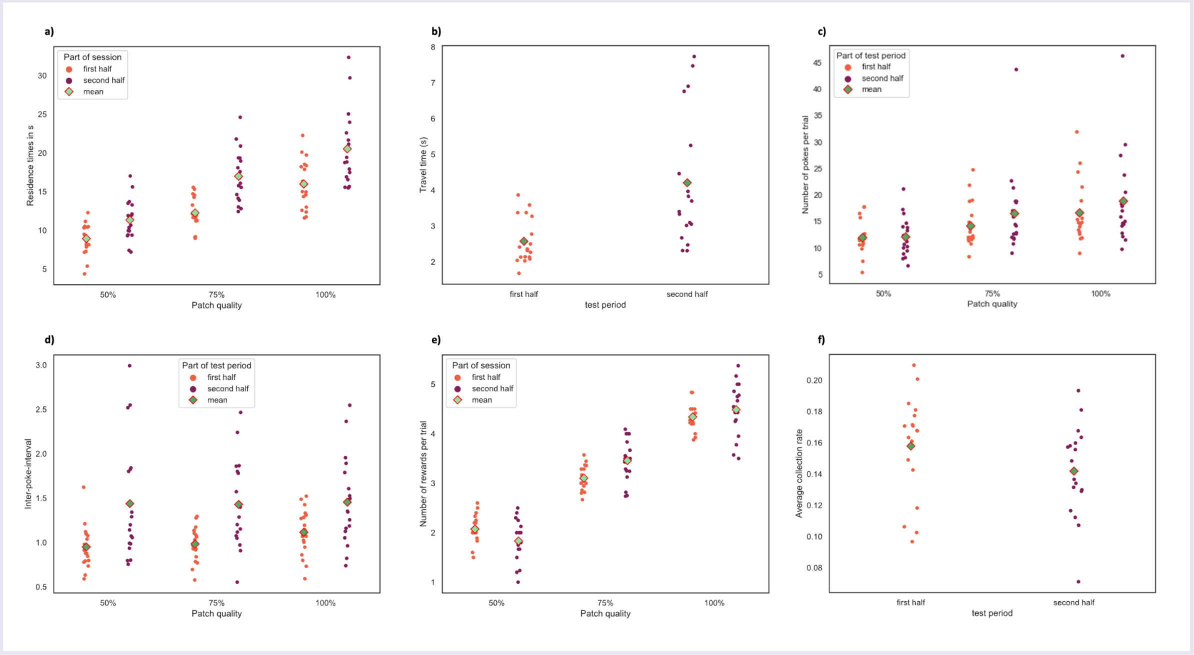

Supplementary Figure 1: **Split-half analysis within sessions**. Residence times (a) as well as travel times (b) increased as a function of session split, likely due to a fading motivation to perform the task as animals got more and more satiated throughout an experimental session. Consistent with this notion, the frequency of nose pokes remained unchanged but the temporal interval between two nose pokes were significantly increased in the second session split (d). Spending more time per patch lead to higher reward yields in medium and high-quality patches but less yield in low-quality patches (e). Altogether, these changes in the animals' behavior led to a significant decrease in the average collection rate (f).

S1.1 Optimal timing of patch-leaving maintained throughout sessions

The decline in task commitment was also reflected in prolonged GUTs in the second half of a session (see Figure 2 a)), [$F(1,17) = 48.193$, $p < .001$], showing that the decreases in the animals' motivation to perform the task, which led to slow non-optimal changes in the task-performance, did also affect the gerbils' patch-leaving behavior with respect to their GUTs. There was no significant effect of patch quality nor evidence for an interaction between session split and patch-quality, suggesting that the effect of the diminishing task commitment was random and affected GUTs equally independent of the the underlying patch-quality [$F(2, 34) = 1.963$, $p = .156$, $F(2, 34) = 0.801$, $p < .457$]. Importantly, consistent with the simple GUT rule, GUTs were constant across patch-qualities also in the second half of a session (see Supplementary Figure 2 a).

Given the increases in GUTs as well as the significant drop in the average collection rate (MCR) in the second compared to the first half of an experimental session (Figure 1 f)), we next tested if the patch departures' alignment with the MVT changed as a function of session duration. The changes in the gerbils GUTs already suggested a further optimization of patch-leaving over time suggesting a continuous optimization of the timing of patch-leaving.

Consistent with this, ICRs at the time of leaving should be lower in the second session split in order to account for the decreased MCR, maintaining a conformity with the MVT prediction of optimal patch-leaving. ICRs at the time of leaving comparable between the two session splits would however indicate that gerbils patch-leaving would become less aligned with the MVT as a function of session duration. In line with the hypothesis that gerbils maintained an optimal timing for patch-leaving throughout the entire experimental session, ICRs at the time of leaving dropped significantly in the second session split [$F(1, 17) = 85.048$, $p < .001$]. Neither the main effect of patch-quality, nor the interaction between session split and patch-quality yielded statistical significance, indicating that the drop in the ICRs was comparable between patch-qualities [$F(1.421, 24.158) = 1.480$, $p = .244$; $F(1.835, 31.197) = 0.484$, $p = .605$]. Thus, accounting for the drop in the MCR, gerbils ICRs were also decreased in the second session split (see Supplementary Figure 2 b). Calculating the difference between ICRs and MCRs, that should be closer to zero in the case of optimal foraging, revealed a further approximation of the two rates in the second session split, indicated by a significant decrease of the ICR-MCR difference [$F(1,17) = 60.689$, $p < .001$]. Again, there was no effect of patch quality, nor a significant interaction [$F(1.421, 24.158) = 1.480$, $p = .244$; $F(1.835, 31.197) = 0.484$, $p = .605$]. This finding shows that the ICRs and MCRs became more aligned as a function of session durations, consistent with optimal foraging according to the MVT (see Supplementary Figure 2 c).

Taken together, these results of the split-half analysis show that behavioral changes indeed suggested a decrease in motivation leading to more task-unrelated behavior. However, central parameters describing the animals patch-leaving behavior suggested that animals continued to optimize the timing of their patch departures despite the fading task-motivation. Importantly, the results do not challenge the conclusion that we made based on the results which were obtained in the analyses where we averaged across entire experimental sessions.

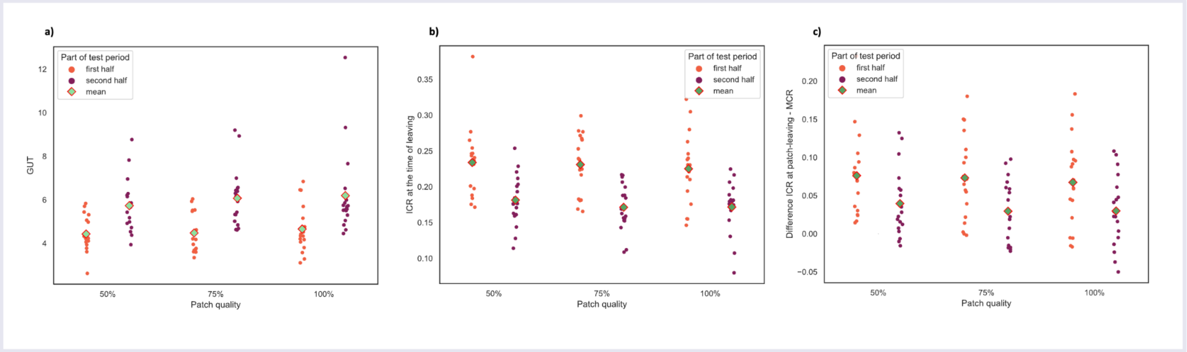

Fig. 2: **Parameters of patch-leaving as a function of session split**. (a) shows the animals' GUTs as function of patch quality for the first and second session split. Consistent with a simple GUT-rule, GUTs did not differ between patch qualities but increased significantly in the second session split. Becoming less engaged with the task over time, estimated ICRs at the time of leaving (b) were significantly decreased in the second session split. However, as also the average collection rate dropped, also the difference between the ICR and MCR at the time of leaving (c) shrank in the second session split so that patch departures were still timed optimally according to the MVT.
